# Tuberculosis in Children and Adolescents, Taiwan, 1996–2003

**DOI:** 10.3201/eid1309.061020

**Published:** 2007-09

**Authors:** Pei-Chun Chan, Li-Min Huang, Yi-Chun Wu, Hsiang-Lin Yang, I-Shou Chang, Chun-Yi Lu, Ping-Ing Lee, Chin-Yun Lee, Luan-Yin Chang

**Affiliations:** * National Taiwan University Hospital and College of Medicine, Taipei, Taiwan; † Center for Disease Control, Taipei, Taiwan; ‡ National Health Research Institutes, Miaoli, Taiwan

**Keywords:** children, tuberculosis, extrapulmonary, Taiwan, BCG, dispatch

## Abstract

Analysis of data from Taiwan’s National Tuberculosis (TB) Registry showed that incidence of TB in persons <20 years of age was 9.61/100,000 person-years, biphasic, and age-relevant, with a major peak in persons slightly >12 years. Aboriginal children were 8.1–17.4× more likely to have TB than non-Aboriginal children.

Because epidemiologic data on childhood tuberculosis (TB) are limited, we conducted a study in Taiwan to estimate the incidence of TB in children and adolescents and to characterize epidemiologic, geographic, and ethnic differences. To do this, we analyzed nationwide data obtained from Taiwan’s National TB Registration, Center for Disease Control.

## The Study

Taiwan’s computer-based system for reporting cases of TB disease was established in 1996. In this system, even suspected cases of TB must be reported and registered. A diagnosis or confirmation of TB is made on the basis of clinical or laboratory findings (*1*). If no TB is confirmed or another diagnosis is made later, the TB registration is cancelled. To ensure compliance with the TB registration system, Taiwan’s National Health Insurance Bureau, a universal healthcare system that has insured 96% of the population since 1996, introduced 2 policies in 1997. The first was the no-notification–no-reimbursement policy, which requires that no claim would be reimbursed for the treatment of a case of TB unless it is reported. The second was the notification-fee policy, which provides an extra cash award to physicians for reporting a new case of TB (*2*).

Population data for Taiwan, including those regarding Aboriginal and non-Aboriginal populations, were obtained from official publications of the Ministry of the Interior (*3*). Age- and gender-specific notification rates (per 100,000) were then calculated based on Taiwan’s National TB Registry data and population data from 1996 through 2003.

Differences in incidences between groups were measured by the χ^2^ test. All reported p values were 2-tailed; p<0.05 was considered statistically significant. The strength of the associations between 2 variables was calculated by using Spearman rank order correlation. All analyses were performed with Epi Info 6.0 (available from www.cdc.gov/epiinfo/epi6/ei6dnjp.htm).

**Figure 1:**
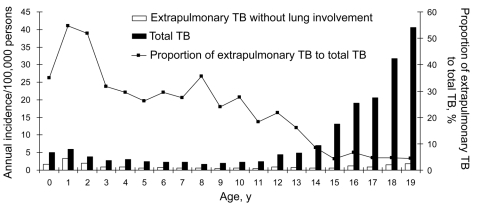
Annual incidence of tuberculosis (TB) and extrapulmonary TB without lung involvement in Taiwanese children, 1996–2003. The line indicates the proportion of extrapulmonary TB without lung involvement to total TB.

Between 1996 and 2003, a total of 5,062 cases were reported, and the overall incidence of TB in patients <20 years of age was 9.6/100,000 person-years, with no significant difference among the years studied (8.2–11.6/100,000 person-years, p = 0.55, with χ^2^ for goodness of fit). Analyzed by age group, incidence of TB for newborns to those 3 years of age was slightly higher than that for those 4–11 years (Figure 1), and incidence increased sharply in children >12 years (p<0.001, χ^2^ for goodness of fit). Analyzed by gender, the male-to-female ratio was 1.32, and boys in the 15- to 19-year-old group were 1.42× more likely than girls to have TB (95% confidence interval [CI] 1.33–1.52, p<0.0001).

We also analyzed the incidence of extrapulmonary TB. Extrapulmonary TB without lung involvement peaked in 1- to 2-year-olds (3.29/100,000 person-years). The distribution was monophasic (Figure 1). The risk of developing extrapulmonary TB was 2.88× higher for children <2 years of age than for children >2 years (95% CI 1.23–6.98, p = 0.012). From 1996 to 2003, the proportion of cases of extrapulmonary TB without lung involvement relative to total TB cases declined with age, from 60% during early childhood to 5% after adolescence We also subdivided the incidence of extrapulmonary TB by site of involvement and the 4 age groups (Table). Bones and joints were the most frequent site of extrapulmonary TB for children <5 years, whereas lymph nodes were the most frequent site for those >5 years.

**Table:** Distribution of different sites of extrapulmonary tuberculosis among 4 age groups, Taiwan, 1996–2003

Site	Age group			
	<4 y (n = 242), no. (%)	5–9 y (n = 96), no. (%)	10–14 y (n = 105), no. (%)	15–19 y (n = 242) no. (%)
Meninges	30 (12)	6 (6)	6 (6)	25 (10)
Lymph nodes	56 (24)	51 (54)	44 (41)	127 (53)
Bone and joint	92 (39)	11(11)	12 (11)	10 (4)
Genitourinary tract	1 (<1)	1 (1)	1 (1)	9 (4)
Skin and eye	15 (6)	3 (3)	6 (6)	10 (4)
Gastrointestinal tract	5 (2)	3 (3)	8 (8)	11 (5)
Others	42 (17)	21 (22)	28 (27)	49 (20)

**Figure 2:**
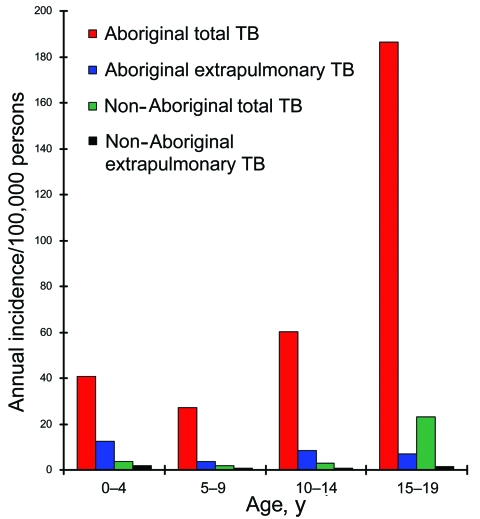
Annual incidence of total tuberculosis (TB) and extrapulmonary TB in aboriginal and non-Aboriginal children, Taiwan, 2000–2003.

The indigenous Aboriginal people of Taiwan represent 1.9% of Taiwan’s population of 22.1 million people (*3*), a proportion similar to that of the aborigines of Australia and Canada. The Aboriginal population in our study had an overall childhood TB incidence of 81.5/100,000 person-years, which was 9.63× (95% CI 3.71–25.04) greater than the incidence for non-Aboriginal children (p<0.0001, χ^2^ test). The ratios of the TB incidence of Aboriginal children to that of non-Aboriginal children were 11.3 in those <4 years old, 13.8 for the 5- to 9-year-old group, 17.4 for the 10- to 14-year-old group, and 8.1 for the 15- to 19-year-old group. Two peaks of extrapulmonary TB occurred in the Aboriginal population (Figure 2), the first in those <4 years of age and the second in those 10–14 years of age.

Geographically, the highest incidence of TB was found in Hualian County, located in eastern Taiwan. This county has a higher proportion of Aboriginal people (28.6%) than any other county in Taiwan. The incidences of TB by geographic area were significantly positively correlated with the percentages of Aboriginal populations for the 4 age groups (r = 0.44, p = 0.03 for those <4 years; r = 0.74, p = 0.00003 for those 5–9 years; r = 0.62, p = 0.0009 for those10–14 years; r = 0.56, p = 0.036 for those 15–19 years, with Spearman rank order correlation).

## Conclusions

In conclusion, the overall incidence of childhood TB is 9.61/100,000 person-years in Taiwan; the incidence is also biphasic and age-relevant, with a major peak found in those just above 12 years of age. The incidence of TB in children is higher in Taiwan than in Western countries (*4*,*5*). In Western countries that do not require bacillus Calmette-Guérin (BCG) vaccination, the highest incidence of childhood TB has been reported in children <5 years of age (*4*,*5*). One possible reason for this difference may be because almost all neonates in Taiwan receive BCG vaccinations (2001, 98%) (*6*), which may protect children <5 years of age from TB.

Previously, higher incidences of TB cases and TB-related deaths have been reported in Aboriginal areas than in non-Aboriginal areas in Taiwan (*1*,*7*). In our study, depending on the age group, the incidence of TB among Aboriginal children was 8.1–17.4× higher than that in non-Aboriginal children. The higher incidence in this population has been attributed to their lower socioeconomic status and an inherited susceptibility (*8*). Aboriginal children may also be exposed to more TB in adults than other groups are or have less access to medical resources than their counterparts (*1*). Although BCG vaccination coverage is high in Taiwan, a lower coverage rate may still play some role in a higher incidence of TB there. Checking the coverage rates of BCG in 2 counties from 2003 through 2005, we found that although 98.5% of all children in Taiwan received BCG vaccinations, only 92.2% of the children in Aboriginal areas did. The difference was significant (p = 0.03, with 2-tailed, 2-proportional *t* test). Therefore, childhood TB in Aboriginal areas might be reduced if the following measures were adopted: implementing directly observed therapy for infected persons, increasing BCG vaccination coverage, and providing more accessible treatment for latent TB infection for the indigenous people in these areas.

We found another peak in incidence of extrapulmonary TB in 10- to 14-year-old Aboriginal children. Although HIV-positive persons were found to have a significantly higher risk for extrapulmonary TB in Arkansas, USA (*9*), we found that none of the Aboriginal people with extrapulmonary TB had reported HIV infection until late 2006. Thus, the increase in extrapulmonary TB in Aboriginal populations is likely related to causes other than HIV and should be investigated further.

In summary, the overall childhood TB incidence was 9.61/100,000 person-years and age-relevant in Taiwan. TB incidence among Aboriginal children was much higher than incidence among non-Aboriginal children. Therefore, efforts to reduce the incidence of childhood TB should be focused on areas with a larger proportion of the Aboriginal population.
